# PReS-FINAL-2065: Oxidative stress is associated to disease activity in a large cohort of JIA at transitional period

**DOI:** 10.1186/1546-0096-11-S2-P77

**Published:** 2013-12-05

**Authors:** J Wipff, C Deslandre, C Gobeaux, A Kahan, D Borderie

**Affiliations:** 1 Rheumatology A, Inserm U1016, Paris, France; 2 Biochemistry, University Paris Descartes, Aphp, Paris, France; 3 Rheumatology A, University Paris Descartes, Aphp, Paris, France

## Introduction

Oxidative damage caused by oxygen free radicals is generally considered a serious mechanism in the pathogenesis of many diseases as cardiovascular diseases, atherosclerosis, and inflammatory rheumatism. Increased oxidative stress has already been described in plasma, saliva and articular fluid of juvenile idiopathic arthritis (JIA) patients. However, most of previous studies did not differentiate ILAR sub-types of JIA and were performed before era of biotherapies.

## Objectives

Our aim was to determine characteristics of blood markers of oxidative stress in a large cohort of JIA at transitional period.

## Methods

One hundred and ten consecutive JIA, fulfilling ILAR criteria, followed in a transition program were included. Age, sex, disease duration, medical or surgical treatments and remission status were collected. Following laboratory tests were performed: ESR, CRP systematically and antinuclear antibodies, rheumatoid factors and anti-CCP when required for the JIA diagnosis. Oxidative stress parameters were: AOPP (Advanced Oxidation Protein Products) and thiols proteins. Control group consisted of twenty healthy controls without inflammatory condition.

## Results

Among the 110 patients' cohort, there were 25 ERA, 16 persistent oligoarthritis, 19 extensive oligoarthritis, 18 polyarticular RF- and 18 RF+ and 14 systemic JIA. Mean age was 21 ± 4 years and mean disease duration was 12.3 ± 3.9 years. Mean global JIA dosage of AOPP was 56.4 ± 47.5 μmol/l and thiols proteins was 473.4 ± 41.6 μmol/l. No differences were detected when comparing JIA to controls for AOPP and thiols (p = 0.4 and p = 0.5, respectively). But, AOPP levels in extensive oligoarthritis sub-group was significantly higher than in controls group (82.6 ± 52.9 vs 46.5 ± 6.5, p = 0.006). Comparison of oxidative stress parameters according to sub-types of JIA showed that extensive oligoarticular sub-groups was associated with higher degree of oxidative stress i.e. higher levels of AOPP compared to ERA and polyarticular JIA and lower levels of thiols proteins compared to ERA.

Thiols proteins levels were strongly associated/correlated with disease activity parameters [remission status (p = 0.008), number of synovitis (p = 0.02), ESR level (p = 0.02) and CRP level (p = 0.0004)].

## Conclusion

Oxidative stress, in this large cohort of JIA patients at transitional period, is tightly associated with disease activity. This confirms that, in JIA, inflammation could lead to articular and/or profound organs damages by oxidative stress. An absolute tight control of JIA activity seems to be primordial for the future (cardiovascular, atherosclerosis) health of the JIA patients. Our results highlight the potential particularity of extensive oligoarticular sub-type of JIA concerning the oxidative stress.

## Disclosure of interest

None declared.

